# Alteration of Inflammatory Mediators in the Upper and Lower Airways under Chronic Intermittent Hypoxia: Preliminary Animal Study

**DOI:** 10.1155/2017/4327237

**Published:** 2017-09-06

**Authors:** Eun Jung Lee, Woon Heo, Joo Young Kim, Hyungchul Kim, Min Jung Kang, Bo Ra Kim, Ji Hyun Kim, Do Yang Park, Chang-Hoon Kim, Joo-Heon Yoon, Hyung-Ju Cho

**Affiliations:** ^1^Department of Otorhinolaryngology, Yonsei University College of Medicine, Seoul, Republic of Korea; ^2^Department of Pharmacology, Yonsei University College of Medicine, Seoul, Republic of Korea; ^3^Department of Chemical and Biomolecular Engineering, Sogang University, Seoul, Republic of Korea; ^4^The Research Center for Human Natural Defense System, Yonsei University College of Medicine, Seoul, Republic of Korea; ^5^The Airway Mucus Institute, Yonsei University College of Medicine, Seoul, Republic of Korea

## Abstract

**Purpose:**

We hypothesized that CIH may affect the upper airway immune system and aimed to verify whether CIH can induce airway inflammation in a murine obstructive sleep apnea (OSA) model.

**Methods:**

C57BL6 male mice were exposed to intermittent hypoxia (CIH group; 5 ~ 21% FiO_2_, 120 sec cycles, 12 h/d, *n* = 6) or room air (Sham group, *n* = 6) for up to 4 weeks in identical chambers. Nasal and lung tissues and lavage fluid were collected and analyzed by multiplex assay. Lung lavage fluid was also utilized for FACS analysis to determine eosinophil count.

**Results:**

We determined the protein level of 24 different cytokines, chemokines, and inflammatory mediators. Among various cytokines, levels of IL-1*α*, IL-1*β*, IL-4, IL-6, and IL-13 were significantly elevated in nose or lung tissue from the CIH group. In addition, MCP-1 and periostin were elevated in nose and lung tissue and lavage fluid from the CIH group.

**Conclusions:**

CIH for 4 weeks altered the levels of inflammatory mediators in both the nose and lungs of mouse model. We suggest that the airway immune system may be deteriorated by CIH and allergic inflammation in the upper or lower airway could be worsened by sleep apnea.

## 1. Introduction

Intermittent hypoxia (IH) is an important factor in the pathophysiology of obstructive sleep apnea syndrome (OSAS). Fletcher et al. developed a system to deliver gases to rats in a cyclic pattern resembling the oxygen profile of severe OSA [1]. OSAS is a disease process caused by repeated episodes of upper airway closure during sleep, resulting in the reduction of airflow and oxygen to the lungs. The oxygen levels can be episodically reduced, which results in the condition of chronic intermittent hypoxia (CIH). CIH may cause an increase in the amount of inflammatory mediators within the bloodstream, which could worsen systemic or local inflammation. Although the evidence of a correlation between airway inflammation and OSAS has been weak, several mechanisms, such as neuromechanical reflex bronchoconstriction, gastroesophageal reflux, and local or systemic inflammation, have been proposed [2].

Recent studies have suggested a relationship between airway inflammation and OSAS. A study conducted in an *in vivo* model of CIH in mice investigated the role of the proinflammatory NF-*κ*B pathway in various tissues and demonstrated increased NF-*κ*B expression in the lungs in response to IH. An association between OSA and asthma has been suggested because many patients with asthma experience asthma attacks during sleep [3–5]. However, it was not known whether nose or lung inflammation is exclusively due to systemic inflammation or results from the activation of specific airway cells. The objective of the present study was to evaluate the effect of CIH on airway inflammatory soluble mediators in an OSA mouse model. We measured levels of cytokines, chemokines, and other soluble mediators from nose and lung tissue and lavage fluid under Sham or CIH conditions.

## 2. Materials and Methods

### 2.1. Animals and Exposure to Chronic Intermittent Hypoxia

This study was performed in strict accordance with the recommendations in the Guideline of the Association for Assessment and Accreditation of Laboratory Animal Care International (AAALAC International) and approved by the Institutional Animal Research Ethics Committee at the Yonsei Medical Center (IACUC Approval number 2013-0299). All experimental protocol methods were carried out in accordance with approved guidelines and were performed on 8-week-old C57BL/6J adult mice. A total of 12 C57BL/6J adult male mice were randomly assigned to the CIH and Sham groups for 4 weeks (*n* = 6 per group). Animals were housed in standard breeding cages and provided with standard mouse chow and water in a temperature- and light-controlled room (22–24°C, 12 h daylight, 12 h darkness). During CIH exposure, mice were transferred from breeding cages to a custom-built CIH chamber coupled with a gas-control delivery system. Gas-control delivery equipment (Live Cell Instrument, Korea) was built to regulate nitrogen and oxygen flow into a customized chamber. For intermittent hypoxia, each 2 min cycle had a 5% nadir of O_2_ followed by restoration of O_2_ to 21% in the chamber. Using infusions of nitrogen into daytime chambers, the CIH groups were subjected to intermittent hypoxia of 5–21% nadir ambient oxygen every 2 minutes. Adult mice show an inverted sleep-wake cycle relative to humans: mice are asleep during the day and are awake at night. Thus, the chronic intermittent hypoxia condition was administered for 12 hours during daytime when mice slept. Also, mice were euthanatized in the morning, but not during the period of hypoxia. The equipment was composed of programmable solenoids and flow regulators to control the inspired oxygen fraction, with timing and magnitude of arterial oxygen desaturation changes as previously reported^1^. Alterations in SpO_2_ of mice in the CIH chamber were monitored with a pulse oxymeter specifically designed for mice (Physiosuite, Kent Scientific©, Connecticut, USA). In the Sham group, all mice were subjected to normoxic conditions in the identical chamber.

### 2.2. Tissue and Lavage Fluid Sampling

To obtain nose or lung mucosal tissue, each mouse was euthanized and its head was resected. Before harvesting tissues, nasal or bronchoalveolar lavage fluid (BAL) was collected. To obtain BAL, 1 mL PBS was instilled and flushed into the trachea using a 1 cc syringe and the lavage fluid was collected. The lungs, including the trachea, were then harvested. To obtain nasal lavage fluid (NAL), 1 mL PBS was instilled into the nasopharyngeal space through the pharyngeal lumen using a 1 cc syringe and PBS flushed from the nostril was collected into an Eppendorf tube. The mouse head was then sagittally sectioned, and mucosal tissue from the nasal cavity was harvested carefully under a microscope.

### 2.3. Magnetic Luminex Screening Assay

To determine the effect of CIH on inflammatory soluble mediators, we analyzed and compared levels of cytokines, chemokines, and some soluble mediators after 4 weeks of hypoxia exposure between the CIH (*n* = 5) and Sham (*n* = 5) groups.

IL-1*α*, IL-1*β*, IL-2, IL-4, IL-5, IL-6, IL-7, IL-13, CCL2 (MCP-1/JE), CXCL-10, MCP-5/CCL12, MDC/CCL22, and periostin were assayed from nasal and lung tissues, NAL, and BAL using the Multiplex human cytokine panel (Millipore, Molsheim, France) and the microassay platform at the Institut de Recherche en Sante, IFR65, Paris, France. Data are expressed as protein concentrations (pg/mL).

### 2.4. Eosinophil Count

The eosinophils in the recovered lung lavage suspensions from the CIH (*n* = 6) and Sham (*n* = 6) groups were counted using a FACS. To prevent nonspecific binding, approximately 2 × 10^6^ cells from harvested lung lavage suspensions were incubated for 5 minutes with a Fc receptor blocking agent (2.4G2) in 100 *μ*L FACS buffer (1× PBS supplemented with 2% heat-inactivated fetal calf serum (Atlanta Biologicals, Norcross, GA) and 0.02% NaN_3_. Next, specific monoclonal antibodies (mAbs) were added to the cell samples and incubated during 45 minutes at 4°C under dark condition. Antibodies purchased from BD Pharmingen (San Diego, CA) were PE-conjugated anti-Siglec-F (E50-2440), PerCP-Cy5.5-conjugated CD45 (30-F11), FITC-conjugated CD11c (HL3), and APC-conjugated CD11b (M1/70) at a final concentration of 1 *μ*g/100 *μ*L. Stained cells were fixed, lysed, then washed, and resuspended in FACS buffer. Stained cell suspensions were analyzed using a Becton Dickinson FACSCalibur flow cytometer (Mountain View, CA) and FlowJo software (Tree Star) [6].

### 2.5. Statistical Analysis

Data are expressed as mean ± standard deviation. The Wilcoxon rank sum test was used to assess differences between nonparametric data. The significance of between-group differences for quantitative variables was determined using the Kruskal-Wallis test followed by the Mann–Whitney *U* test. All statistical analyses were performed using the statistical software package SPSS 20.0 (IBM Inc., IL, USA). Statistical significance was set below a value of 0.05.

## 3. Results

### 3.1. CIH-Induced Inflammatory Cytokine Response

**Figure 1:**
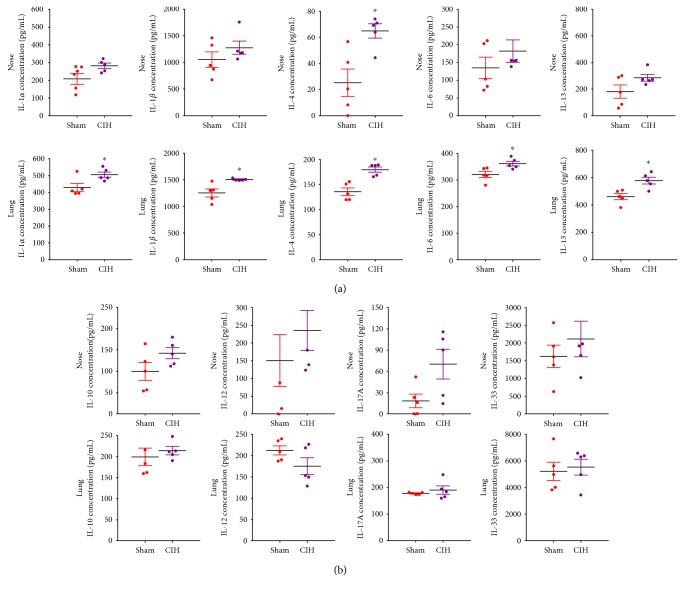
Changes in inflammatory cytokine responses. (a) Levels of IL-1*α*, IL-1*β*, IL-4, IL-6, and IL-13 from nose tissue were markedly increased by exposure to chronic intermittent hypoxia (CIH) compared to those of the Sham group. The level of IL-4 from nose tissue showed the only statistically significant difference. CIH exposure significantly increased the release of IL-1*α*, IL-1*β*, IL-4, IL-6, and IL-13 from the lung tissue. (b) The level of IL-10, IL-12, IL-17A, and IL-33 showed no statistical difference between the two conditions. ^∗^*p* < 0.05 was considered as statistically significant compared to the sham groups.

CIH-induced inflammatory cytokines were detected in nose and lung tissue but barely detected in NAL or BAL samples. In the nose, the levels of IL-1*α*, IL-1*β*, IL-4, IL-6, and IL-13 were markedly increased in the CIH group compared to those in the Sham group. Among them, IL-4 was significantly elevated after 4 weeks of CIH in the nasal tissue (CIH versus Sham = 64.99 ± 12.28 pg/mL versus 25.30 ± 23.38 pg/mL, *p* < 0.05, Figure 1(a)). Meanwhile, in the lung, CIH exposure significantly increased the levels of IL-1*α*, IL-1*β*, IL-4, IL-6, and IL-13. No differences were observed between the CIH and Sham groups in levels of IL-10, IL-12, IL-17A, and IL-33 (Figure 1(b)). IL-2, IL-5, and IL-7 were not detected in any samples.

### 3.2. CIH-Induced Inflammatory Chemokine Response

**Figure 2:**
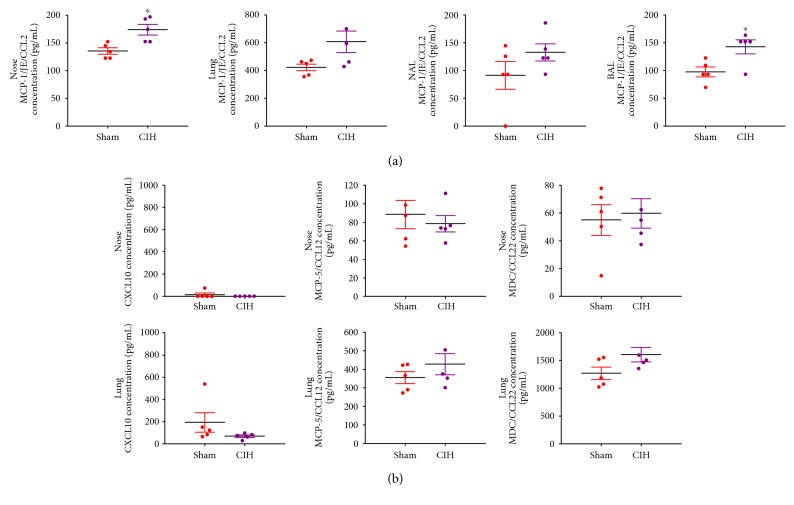
Changes in inflammatory chemokine responses. (a) Increased levels of CCL2 (MCP-1/JE) were noted in nose, lung, nasal lavage fluid (NAL), and bronchoalveolar lavage fluid (BAL) in chronic intermittent hypoxia (CIH). A significant difference between Sham and CIH groups was noted with both nose tissue and BAL. (b) No differences were observed between Sham and CIH treatment in terms of CXCL-10, MCP-5/CCL12, and MDC/CCL22. ^∗^*p* < 0.05 was considered as statistically significant compared to the sham groups.

Increased levels of CCL2 (MCP-1/JE) were noted in nose, lung, NAL, and BAL samples after exposure to CIH (Figure 2(a)). Among the samples, nasal tissue and BAL showed statistically significant elevation of CCL2 in the CIH group. No differences were observed between the Sham and CIH groups in terms of CXCL-10, MCP-5/CCL12, or MDC/CCL22 (Figure 2(b)).

### 3.3. Changes in Periostin

**Figure 3:**
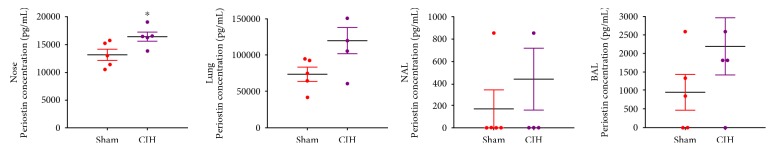
Changes in periostin levels. The level of periostin was markedly increased by exposure to chronic intermittent hypoxia (CIH), compared to that of the Sham group, in all samples. Among them, periostin in the nose tissue was significantly elevated by 4 weeks of CIH. ^∗^*p* < 0.05 was considered as statistically significant compared to the sham groups.

The level of periostin was markedly increased by exposure to CIH, compared to that of the Sham group, in all samples. Among them, periostin was significantly elevated after 4 weeks of CIH in the nasal tissue (CIH versus Sham = 16437.98 ± 1838.53 pg/mL versus 13175.76 ± 2273.97 pg/mL, *p* < 0.05, Figure 3).

### 3.4. Changes in Eosinophil Count

**Figure 4:**
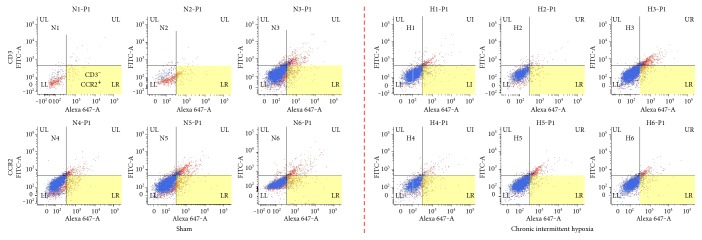
Eosinophil counts from bronchoalveolar lavage fluid (BAL). There was no significant difference in eosinophil count between the chronic intermittent hypoxia (CIH) and Sham groups.

Because the elevation of Th2 cytokines, such as IL-4 and IL-13, was noted after CIH exposure, we determined the eosinophil count in the BAL samples. We counted CD3^−^CCR2^+^ cells as eosinophils among granulocyte populations by FACS analysis. There was no significant difference in eosinophil count between the two groups (Figure 4).

## 4. Discussion

OSAS is a common medical condition that is being increasingly recognized as an important cause of medical morbidity or mortality. The pathogenesis of OSAS, while not completely understood, is likely due to the interaction between unfavorable anatomic upper airway susceptibility and sleep-related changes in upper airway function [7]. OSAS is characterized by repeated episodes of upper airway occlusion that result in brief periods of breathing cessation (apnea) or a marked reduction in respiratory flow (hypopnea) during sleep. This respiratory pattern is accompanied by a CIH condition, which can cause overt inflammatory insult characterized by increased serum concentrations of cytokines or chemokines [7]. Repetitive hypoxia events seen in OSAS likely lead to oxidative stress and the generation of reactive oxygen species, which may play an important role in activating inflammatory responses in patients with OSA [8].

Recent studies have suggested a relationship between airway inflammation and OSAS. Asai et al. verified the increased secretion of cytokines in sputum, which was related to the severity of OSAS [9]. Inflammatory and oxidative stress markers, including pentane, exhaled nitric oxide, IL-6, and 8-isoprostane, were other mediators detected in the expired breath of patients with OSAS, and this may reflect the possibility of airway inflammation in the status of OSA [10, 11]. Among various airway diseases, it is widely recognized that asthma attacks often occur at night and up to 90% of patients with asthma are awakened by breathing difficulty at least once a week [3–5]. Therefore, effective treatment of OSA has been suggested to reduce nocturnal symptoms in patients with asthma who also have sleep breathing disorder [12]. Accordingly, IH for 35 days induced overexpression of VEGF, HIF-1*α*, iNOS, NF-*κ*B, and TNF-*α* in mouse lungs [13]. In an experiment using primary nasal epithelial cells exposed to intermittent hypoxia for 24 h, increased levels of MMP-2, MMP-9, IL-8, PDGF-AA, and VEGF were observed [14]. These studies imply an interesting association of pathophysiology between sleep apnea and allergic airway diseases, but there is still a lack of evidence confirming their linkage. Therefore, we hypothesized that CIH may potentially affect the upper and lower airway immune system.

Although our screening did not include all cytokines or chemokines, we identified increased levels of IL-1*α*, IL-1*β*, IL-4, IL-6, and IL-13 in nose and lung tissue in the CIH group compared to those of the Sham group. Moreover, the level of CCL2 (MCP-1/JE) was more markedly increased by CIH exposure compared to Sham conditions. We noted that levels of Th2-derived cytokines, such as IL-4 and IL-13, were elevated in both nose and lung tissues. Furthermore, we evaluated the periostin level and newly discovered that its level was augmented by CIH. Periostin acts on *α*v integrin on keratinocytes, inducing production of proinflammatory cytokines including TSLP [15]. Periostin is an extracellular matrix protein that is induced by IL-4 or IL-13 in airway epithelial cells and lung fibroblasts [16]. IL-4, IL-13, periostin, and proinflammatory cytokines including TSLP act to amplify Th2 immune responses resulting in allergic airway diseases. Our results show that the periostin level was markedly increased by CIH in all nose, lung, NAL, and BAL samples. We have no evidence that the elevation of periostin was associated with the increase of IL-4 or IL-13, but our observation implies that periostin could be involved in the amplification, or persistence, of airway inflammation by CIH.

To confirm the involvement of allergic pathophysiology in sleep apnea, we also determined the eosinophil count from the BAL samples; Th2-derived cytokines, together with eotaxin, play a critical role in the induction of airway hyperreactivity and the development of chronic airway wall remodeling [17]. However, contrary to our expectation, we observed no significant difference in eosinophil count between the Sham and CIH groups. This result is consistent with that of Broytman et al., showing that eosinophil count was not altered by CIH [18].

Meanwhile, no differences were observed between the CIH and Sham groups in terms of IL-10, IL-12, IL-17A, IL-33, CXCL-10, MCP-5/CCL12, and MDC/CCL22. Moreover, IL-2, IL-5, and IL-7 were not detected in any samples. Hogan et al. demonstrated that fibrotic changes in the epithelium after chronic allergen inhalation were independent of IL-5 in sensitized BALB/c mice [19]. These results suggest that eosinophil or IL-5 activation could be less critical phenomena in OSA condition.

However, we assume that the symptoms of patients with allergies could be aggravated in the presence of OSA, because Broytman et al. showed that 30 days of CIH exposure in a rat model resulted in an altered airway immune response [18]. Using an allergen-induced allergic asthma rat model, they found that CIH increased the collagen density around larger airways resulting in worsening of pulmonary function. A distinguishing point of our study is that we screened various mediators from the nose and lungs in a mouse model and provided potential evidence of the pathophysiology of OSA-induced airway inflammation. Thus, additional studies using allergic rhinitis or asthma mouse models under CIH conditions would be very interesting and could reveal more clearly the consequences of OSA in allergic airway diseases.

The main drawback of this study was the inability to show statistically significant differences in some inflammatory responses because of the limited sample size. A larger sample size would be needed to reinforce this result. The rat physiological study showed that the weight loss was noted in the hypoxic groups compared to the normoxic groups and weight change also induces inflammation [20]. Therefore, the change of weight loss by CIH condition should also be considered to verify the change of inflammatory soluble mediators by CIH for future study. However, our study is valuable, in that, it is the first study to verify the influence of CIH on the airways by determining inflammatory mediators in an *in vivo* mouse model.

In summary, we found that IL-4, IL-13, CCL2 (MCP-1/JE), and periostin levels were augmented by CIH and these may be involved in the pathophysiology of allergic airway diseases under OSA conditions. We expect that patients with preexisting allergic airway diseases could be vulnerable to CIH and their symptoms could easily be intensified by comorbid sleep apnea.

## 5. Conclusion

We objectively confirmed that CIH, which is one of the main factors in the pathogenesis of OSA, induced inflammatory responses in the airways of a murine OSA model. CIH treatment for 4 weeks altered the level of various cytokines or chemokines, notably including IL-4, IL-13, CCL2, and periostin, in nose or lung tissues. Therefore, the airway immune system could be deteriorated in patients with OSA, and a vicious cycle between OSA and airway inflammation could be repeated under repetitive sleep apnea. We suggest that if allergic asthma or rhinitis is combined with OSA, treatment of OSA would be beneficial to lessen the possible priming or worsening of allergic symptoms by OSA.
